# Comparison of first‐tier whole‐exome sequencing with a multi‐step traditional approach for diagnosing paediatric outpatients: An Italian prospective study

**DOI:** 10.1002/mgg3.2316

**Published:** 2023-12-02

**Authors:** Erica Rosina, Lidia Pezzani, Erika Apuril, Laura Pezzoli, Daniela Marchetti, Matteo Bellini, Camilla Lucca, Camilla Meossi, Marta Massimello, Milena Mariani, Agnese Scatigno, Elisa Cattaneo, Lorenzo Colombo, Silvia Maitz, Anna Cereda, Donatella Milani, Luigina Spaccini, Maria Francesca Bedeschi, Angelo Selicorni, Maria Iascone

**Affiliations:** ^1^ Laboratory of Medical Genetics ASST Papa Giovanni XXIII Bergamo Italy; ^2^ Paediatric Unit ASST Papa Giovanni XXIII Bergamo Italy; ^3^ Fondazione IRCCS Ca’ Granda Ospedale Maggiore Policlinico Milan Italy; ^4^ Department of Pediatrics Fondazione IRCCS San Gerardo dei Tintori Monza Italy; ^5^ Department of Pediatrics ASST Lariana Sant' Anna Hospital Como Italy; ^6^ Clinical Genetics Unit, Department of Obstetrics and Gynecology V. Buzzi Children's Hospital, University of Milan Milan Italy; ^7^ Neonatal Intensive Care Unit (NICU) Fondazione IRCCS Ca' Granda Ospedale Maggiore Policlinico Milan Italy; ^8^ Medical Genetics Service IOSI, Ente Ospedaliero Cantonale Lugano Switzerland; ^9^ Clinical Genetics Unit Fondazione IRCCS Ca' Granda Ospedale Maggiore Policlinico Milan Italy

**Keywords:** delayed diagnosis, genetic testing, paediatric patients, traditional approach, whole exome sequencing

## Abstract

**Background:**

The recent guidelines suggest the use of genome‐wide analyses, such as whole exome sequencing (WES), at the beginning of the diagnostic approach for cases with suspected genetic conditions. However, in many realities it still provides for the execution of a multi‐step pathway, thus requiring several genetic tests to end the so‐called ‘diagnostic odyssey’.

**Methods:**

We reported the results of GENE Project (Genomic analysis Evaluation NEtwork): a multicentre prospective cohort study on 125 paediatric outpatients with a suspected genetic disease in which we performed first‐tier trio‐WES, including exome‐based copy number variation analysis, in parallel to a ‘traditional approach’ of two/three sequential genetic tests.

**Results:**

First‐tier trio‐WES detected a conclusive diagnosis in 41.6% of patients, way above what was found with routine genetic testing (25%), with a time‐to‐result of about 50 days. Notably, the study showed that 44% of WES‐reached diagnoses would be missed with the traditional approach. The diagnostic rate (DR) of the two approaches varied in relation to the phenotypic class of referral and to the proportion of cases with a defined diagnostic suspect, proving the major difference for neurodevelopmental disorders. Moreover, trio‐WES analysis detected variants in candidate genes of unknown significance (*EPHA4*, *DTNA*, *SYNCRIP*, *NCOR1*, *TFDP1*, *SPRED3*, *EDA2R*, *PHF12*, *PPP1R12A*, *WDR91*, *CDC42BPG*, *CSNK1D*, *EIF3H*, *TMEM63B*, *RIPPLY3*) in 19.4% of undiagnosed cases.

**Conclusion:**

Our findings represent real‐practice evidence of how first‐tier genome‐wide sequencing tests significantly improve the DR for paediatric outpatients with a suspected underlying genetic aetiology, thereby allowing a time‐saving setting of the correct management, follow‐up and family planning.

## 1 INTRODUCTION

Although individually rare, when taken together genetic diseases have a great impact in terms of social and public health burden, with an estimated prevalence ranging from 3.5% to 5.9% of the world population (i.e. about 1 in 20 people) (NguengangWakap et al., 2020). Since approximately 70% of the genetic conditions have a paediatric onset (NguengangWakap et al., 2020), an accurate definition of genetic aetiology is essential for these patients in order to tailor the correct management, follow‐up and family counselling. To date, traditional diagnostic approach for patients with suspected genetic conditions usually provides for the execution of a sequence of genetic tests (such as chromosomal microarray (CMA), single‐gene analyses, and targeted panels of genes). Nevertheless, the rarity of these conditions, their variability in clinical expression and genetic heterogeneity, combined with the partial overlap of many clinical pictures and the possible lack of age‐related markers, make this hierarchical diagnostic pathway time‐consuming, expensive and often ineffective, requiring several genetic tests to end the so‐called ‘diagnostic odyssey’.

Next‐generation sequencing (NGS) technologies have completely revolutionized the genetic diagnostic approach, enabling rapid sequencing of genes with cost–time effectiveness (Koboldt et al., 2013). Compared to its application to targeted gene panel, whole‐exome sequencing (WES) has the advantage of simultaneously sequencing the entire coding genome and filtering variants with an unbiased approach according to their effects on protein and transcript, their consistency with patient's phenotype and the suspected inheritance model.

The overall diagnostic rate of WES is extremely variable based on the analysed population, the use of a singleton versus trio‐WES (i.e. analysis of the proband and his parents) and the experience of the laboratory, being estimated between 28% and 68% (Malinowski et al., 2020; Souche et al., 2022). Moreover, specific algorithms developed to call copy number variants (CNVs) with exome‐based data (de Ligt et al., 2013; Tan et al., 2014) are increasingly being used, therefore expanding the diagnostic potential of WES analysis. For these reasons, the American College of Medical Genetics and Genomics (ACMG) now recommends WES or whole genome sequencing (WGS) as first or at least second‐tier test in patients with one or more congenital anomalies, developmental delay or intellectual disability (Manickam et al., 2021).

However, except for its growing use as first‐tier test in critically ill infants of intensive care units (Australian Genomics Health Alliance Acute Care Flagship, 2020; Kingsmore et al., 2019; Muriello and Basel, 2022; Stark et al., 2017), in many realities, WES is still performed at the end of a multi‐step diagnostic approach on outpatients with suspected genetic conditions. Several factors, like the perception by clinicians of higher costs and referral time, the concern of detecting more variants of uncertain significance, up to the relatively low number of laboratories performing WES, may contribute to the limited use of exome as a first‐tier test in these patients.

Here we report the results of GENE Project (Genomic analysis Evaluation NEtwork) whose aim was to compare the diagnostic yield and implications of first‐tier trio‐WES including exome‐based CNV analysis to a traditional diagnostic approach, in paediatric outpatients with a suspected genetic condition.

## 2 SUBJECTS AND METHODS

### 2.1 Participants

From June 2019, a total of 125 subsequent recruitable probands and their biological parents were prospectively enrolled by five different public hospitals across Lombardy (Italy). Patients were recruited in accordance with the following inclusion criteria: (1) neonatal/paediatric age (≤18 years old); (2) outpatient setting; (3) clinical pictures suggestive of a genetic aetiology. Probands were excluded in the subsequent scenarios: (1) the proband already underwent other genetic tests (except for foetal karyotype performed for maternal age above 35 years old); (2) suspect of a chromosomal aneuploidy syndrome or a condition detectable by the Newborn Screening proposed in Lombardy (Ruoppolo et al., 2022); (3) clinical diagnosis of a genetic condition not identifiable with WES technology (for example, methylation disorders or repeat expansion diseases); (4) biological parents not available for testing.

For all the patients, detailed clinical evaluation and medical/familial history with comprehensive pre‐test counselling were undertaken by clinical geneticists.

Patients' clinical profile was then classified in: (1) malformation syndrome ± intellectual disability (ID) (i.e. one congenital malformation + ID or ≥2 congenital malformations); (2) neurodevelopmental disorder (NDDs) (i.e. psychomotor retardation (RPM)/ID + dysmorphisms or neurological anomalies ± ID); (3) connective tissue disease; (4) metabolic disorder; (5) skeletal dysplasia; (6) growth disorder and (7) hematologic/immunological disease.

At recruitment, the clinical geneticist indicated the possible presence of a suspected diagnosis and a series of two/three genetic tests that would be sequentially performed as ‘traditional approach’ (both unknown by the laboratory that performed trio‐WES analyses): the alternative diagnostic pathway has been run in parallel in the referring hospitals.

Trio‐WES has been performed at the Medical Genetics Laboratory of ASST Papa Giovanni XXIII of Bergamo as part of the GENE Project. Written informed consent for the Project recruitment and the trio‐WES analysis was obtained from the parents of the patients.

### 2.2 Exome sequencing analysis and report

Genomic DNA was extracted from peripheral blood samples of probands and parents using standard procedures. The exonic regions and flanking splice junctions of the genome were captured using the Clinical Research Exome v.2 kit (Agilent Technologies, Santa Clara, CA, USA). Sequencing was performed on a NextSeq500 Illumina system with 150bp paired‐end reads. Reads were aligned to human genome build GRCh37/UCSC hg19 and analysed for sequence variants using a custom‐developed analysis tool, already validated and standardized (Pezzani et al., 2018). The variant call file, including single nucleotide polymorphism and indels, was annotated by querying population frequencies databases and mutation databases, including the Genome Aggregation Database (http://gnomad.broadinstitute.org/), ClinVar (https://www.ncbi.nlm.nih.gov/clinvar/) and Human Gene Mutation Database Professional (HGMD, Release 2017.4). To prioritize variants, a sequential filtering strategy was applied, retaining only variants with the following characteristics: (a) potential effect on protein and transcript (splicing, missense, nonsense and frameshift); (b) consistency with the patient's phenotype according to the Human Phenotype Ontology classification (www.human‐phenotype‐ontology.org/); (c) consistency with the suspected inheritance model (X‐linked, autosomal recessive or de novo) with a frequency in the general population compatible with prevalence and incidence of the disease and showing a pathogenic mechanism corresponding to the one expected for the disease (Pezzani et al., 2018). Variants were classified based on ACMG guidelines (Richards et al., 2015). The potential causative variants were subsequently confirmed by Sanger sequencing in the proband and parents using an independent DNA sample.

Two pipelines were used to identify the copy number variants based on ExomeDepth and one created in‐house, as previously described (Pezzoli et al., 2021). All the CNVs detected by both pipelines were annotated with the genes involved and related diseases and classified according to ACMG and the Clinical Genome Resource (ClinGen) guidelines (Riggs et al., 2020).

De novo or bi‐allelic variants in genes of unknown significance (GUS) were recorded only in cases of genes undergoing validation or candidate genes with multiple data supporting a potential causative role for the patient's clinical picture (such as the presence of matching patient in international databases as GeneMatcher (https://genematcher.org/statistics/)).

### 2.3 Data collection

After the execution of trio‐WES analysis and alternative diagnostic pathway for all the patients, data were collected, including sex, age at the time of recruitment, family history, detailed clinical characterization using standard HPO (Human Phenotype Ontology) terminology and presence of a clinical suspect.

The results of the first‐tier WES and the traditional approach (i.e. the first and eventually the second genetic test indicated at the time of recruitment) were reviewed. The diagnostic rates (DRs) of the two alternative pathways (as the amount of ‘pathogenic’ or ‘probably pathogenic’ results on the total exams), as well as the proportion of uncertain outcomes, were then compared, in association with clinical profiles and classification of diseases.

For statistical comparison between the DRs of the two approaches, the McNemar's test was used. A *p* < 0.05 (two‐sided) was considered statistically significant.

## 3 RESULTS

### 3.1 Characteristics of the cohort

Of the 125 recruited paediatric outpatients, 75 were males (60%) and 50 were females (40%). Due to the criteria mentioned above, all individuals included were under 18 years of age at the time of enrolment: the average age was 5 years (range 1 month–17 years), while the median age was 3 years 7 months.

Dysmorphisms and ID/RPM were the most reported phenotypic features, respectively, in 52.8% and 47.2% of patients, followed by central nervous system functional or structural anomalies (in 39.2%), skeletal (in 25.6%) or growth anomalies (in 23.2%).

Based on clinical phenotype, the patients have been classified as (1) malformation syndromes in 23.2% of cases (29/125); (2) neurodevelopmental disorders (NDD) in 50.4% (63/125); (3) connective tissue diseases in 6.4% (8/125); (4) metabolic diseases in 3.3% (4/125); (5) skeletal dysplasias in 12.0% (15/125) and as (6) growth anomalies in 4.8% (6/125) of cases. A specific condition or a group of related disorders was clinically suspected in approximately 31.2% (39/125) of patients. The proportion of patients with a suspected condition varies widely among the most represented phenotypic classes, with 53.3% (8/15) in skeletal dysplasia patients, 44.8% (13/29) in malformative syndromes to only 15.9% (10/63) in NDD patients.

### 3.2 Results of first‐tier WES approach

Trio‐WES analysis coupled with CNVs calling yielded an overall diagnostic rate of 41.6%, that is, in 52 patients the analysis identified pathogenic/likely pathogenic variants causative of the patient's clinical picture (Supplementary Table S1). The definite result was available to the clinician in 52 days on average (range 13–183 days).

Among the conclusive trio‐WES analyses, 65.4% (34/52) had causal variants associated with autosomal dominant disorders, the majority of which were de novo except two that were inherited from an affected parent, 23% (12/52) had autosomal recessive conditions and 5.8% (3/52) had variants related to X‐linked dominant or recessive disorders. In three patients (5.8%), WES allowed the detection of a chromosomal aneuploidy: in particular a trisomy 13, a Turner syndrome and a patient with Klinefelter syndrome and an associated PTPN11‐related Noonan syndrome (OMIM #163950). In addition, exome‐based CNV analysis revealed one patient with a 6 Mb‐duplication of chromosome 9p that subsequently resulted in a trisomy 9p at CMA.

The trio‐WES result was inconclusive only in one patient (0.8% of the total cases). Indeed, a de novo variant of unknown significance (VUS) was identified in the *WDR26* gene (OMIM *617424).

### 3.3 Potential diagnoses identified with trio‐WES

Among patients who tested negative for trio‐WES, 19.4% (14/72) presented variants in GUS or candidate genes (Table 1). One of these cases showed ‘de novo’ variants in two different candidate genes, potentially co‐responsible for the clinical picture.

**TABLE 1:** Summary of patients with variants in GUS genes.

Case	Sex	Age	Clinical picture	Gene	Variant, inheritance	Diagnosis by ‘traditional approach’
1	M	5 months	Hypotonia, reduced spontaneous motility, dysmorphisms, micrognathia, unilateral persistent hyperplastic primary vitreous, overgrowth	*EPHA4* OMIM *602188	NM_001304536.2:c.935G>T p.Gly312Val, htz *de novo*	No
6	M	2 years 6 months	Developmental delay, epilepsy, aspecific brain anomalies, dysmorphisms, corneal leukoma	*DTNA* OMIM *601239	NM_001390.4:c.1670G>T p.Gly557Val, htz *de novo*	Yes, MidXq28 duplication syndrome
18	F	3 years 9 months	Moderate global developmental delay, sialorrhea, dysmorphisms	*SYNCRIP* OMIM *616686	NM_006372.5:c.787_790del p.Phe263fs, htz class 5 after publication (Gillentine et al., 2021) *de novo*	No
31	M	10 years 8 months	Moderate intellectual disability, EEG anomalies, dysmorphisms	*NCOR1* OMIM *6008491	NM_006311.4:c.4243del p.Arg1415fs, htz mat (mosaic)	No
48	M	2 years 6 months	Developmental delay, hydrocephalus, aspecific brain anomalies, dysmorphisms, macrocephaly, overweight, recurrent fevers	*TFDP1* OMIM *189902	NM_007111.5:c.500G>A p.Arg167Gln, htz *de novo*	No
54	F	4 years 8 months	Moderate developmental delay, autism	*SPRED3* OMIM *609293	NM_001042522.3:c.1229G>T p.Arg410Leu, htz *de novo*	No
56	M	9 years	Intellectual disability, ADHD, epilepsy, macrocephaly	*EDA2R* OMIM *300276	NM_021783.5:c.349C>T p.Gln117ter, hemizygous mat	No
58	F	5 years 1 month	Dysmorphisms, strabismus, VSD and bicuspid aortic valve, umbilical hernia, brachydactyly	*PHF12* OMIM *618645	NM_001033561.2:c.301C>T p.Arg101Trp, htz *de novo*	No
				*PPP1R12A* OMIM *602021	NM_002480.3:c.232C>T p.His78Tyr, htz *de novo*	
75	M	3 years 5 months	Severe developmental delay, microcephaly, severe microlissencephaly, agenesis of corpus callosum, epilepsy, spastic tetraparesis, laryngomalacia, bicuspid aortic valve, congenital hip dislocation, growth retardation, dysmorphisms	*WDR91* OMIM *616303	NM_014149.4:c.511+1A>G, hmz mat/pat	No
84	M	10 years 11 months	Mild intellectual disability, ADHD, epilepsy and EEG anomalies, 4th‐5th finger clynodactyly	*CDC42BPG* OMIM *613991	NM_017525.3:c.1128dup p.Thr377fs, htz *de novo*	No
96	M	7 years 8 months	Intellectual disability, autism spectrum disorder, strabismus, Ebstein anomaly and ASD, intestinal invagination, dysmorphisms	*CSNK1D* OMIM *600864	NM_001893.6:c.672A>C p.Lys224Asn, htz *de novo*	No
104	F	6 years 10 months	Intellectual disability, microcephaly, dysmorphisms	*EIF3H* OMIM *603912	NM_003756.3:c.391C>T p.Arg131Trp, htz pat NM_003756.3:c.457+1G>C, htz mat	No
105	M	5 months	Developmental delay, partial agenesis of the corpus callosum, visual impairment, hyperbilirubinaemia	*TMEM63B* OMIM *619952	NM_018426.3:c.1738G>A p.Gly580Ser, htz *de novo*	No
116	M	3 months	Axial hypotonia, polymicrogyria, dolichocephaly, dysmorphisms	*RIPPLY3* OMIM *609892	NM_018962.3:c.375del p.Glu125fs, htz *de novo*	No

Abbreviations: ADHD, attention deficit hyperactivity disorder; ASD, atrial septal defect; EEG electroencephalogram; hmz, homozygous; htz, heterozygous; mat, maternal; pat, paternal; VSD, ventricular septal defect.

Of the 15 GUSs encountered, two have recently become disease genes: particularly, *PPP1R12A* gene has been associated with Genitourinary and/or brain malformation syndrome (OMIM #618820) (Hughes et al., 2020), while *SYNCRIP* gene is implicated in a new neurodevelopmental disorder (Gillentine et al., 2021). For most of the remaining GUSs, that is, eight out of 15, international matching databases have paved the way for collaborative studies to investigate their potential role in genetic diseases.

Nearly 66.7% (10/15) of GUSs were identified in patients with neurodevelopmental disorder, particularly in 16% (10/63) of the total NDD cases; followed by 13.3% of GUS detection in patients with skeletal dysplasia and 10.3% in cases classified as malformation syndromes.

### 3.4 Results of traditional approach

Regarding the conventional approach, different types of tests were carried out: CMA, MLPA (Multiplex Ligation‐dependent Probe Amplification) of single genes, *FMR1* analysis, single gene sequencing, gene panels sequencing and clinical exomes.

The overall diagnostic rate of traditional pathway was 24.8% (31/125); among these diagnoses, 51.6% (16/31) were obtained with the first proposed test, while 42% (13/31) with the second one and 6.5% (2/31) by a third one. Especially, CMA was listed as first‐tier test in 40.2% (52/125) of probands, and as second‐tier test in 20.8% (26/125) of them: its diagnostic yield in our cohort resulted 2.5% (2/78).

### 3.5 Diagnostic rates comparison between trio‐WES and traditional pathway

The overall DR of our cohort was significantly improved by trio‐WES approach (*p* value <0.001). In 31.2% of cases with specific clinical suspicion, the hypothesized diagnosis was confirmed with both approaches only in 33.3% (13/39) of patients. However, the WES‐trio detected diseases other than suspected in an additional 20.5% (8/39) of cases. Even considering only patients with clinical suspicion, trio‐WES was significantly superior in terms of DR (*p* = 0.008). The DR of the two approaches varies in relation to the phenotypic categories and the proportion of patients with a diagnostic suspicion: for the clinical classes with more than 10 patients, a comparison between the DR of the two approaches is reported in Table 2.

**TABLE 2:** Comparison between detection rates of the two approaches, in relation to the phenotypic class and the proportion of patients with clinical suspicion.

	% of patients with a clinical suspect	Trio‐WES			Traditional approach		
		DR in pt with clinical suspect	DR in pt without clinical suspect	Overall DR	DR in pt with clinical suspect	DR in pt without clinical suspect	Overall DR
Malformation Syndrome (no. 29)	44.8% (13/29)	69.2% (9/13)	43.8% (7/16)	55.2% (16/29)	61.5% (8/13)	31.3% (5/16)	44.8% (13/29)
Neurodevelopmental disorder (no. 63)	15.9% (10/63)	60% (6/10)	32.1% (17/53)	36.5% (23/63)	20% (2/10)	11.3% (6/53)	12.7% (8/63)
Skeletal dysplasia (no. 15)	53.3% (8/15)	25% (2/8)	71.4% (5/7)	46.7% (7/15)	25% (2/8)	42.9% (3/7)	26.7% (4/15)

Abbreviations: DR, diagnostic rate; pt, patients.

Trio‐WES could identify all the ‘traditional approach’ diagnoses except for two cases (i.e. the 1.6% of patients): in particular, a male patient presented a pathological expansion of *FMR1* gene promoter (OMIM #300624) and the second one was diagnosed with a maternal MidXq28‐duplication syndrome (OMIM #300815). Another patient, negative both at first‐tier WES and at the indicated traditional pathway, successively underwent karyotype on fibroblasts that evidenced low‐level mosaicism (9%) for trisomy 8. Hence, only 2.4% (3/125) of the probands received a diagnosis not pinpointed by trio‐WES analysis.

On the contrary, about half of the diagnoses detected by trio‐WES, in particular 44.2% of them, would be missed using the conventional diagnostic pathway.

## 4 DISCUSSION

**FIGURE 1:**
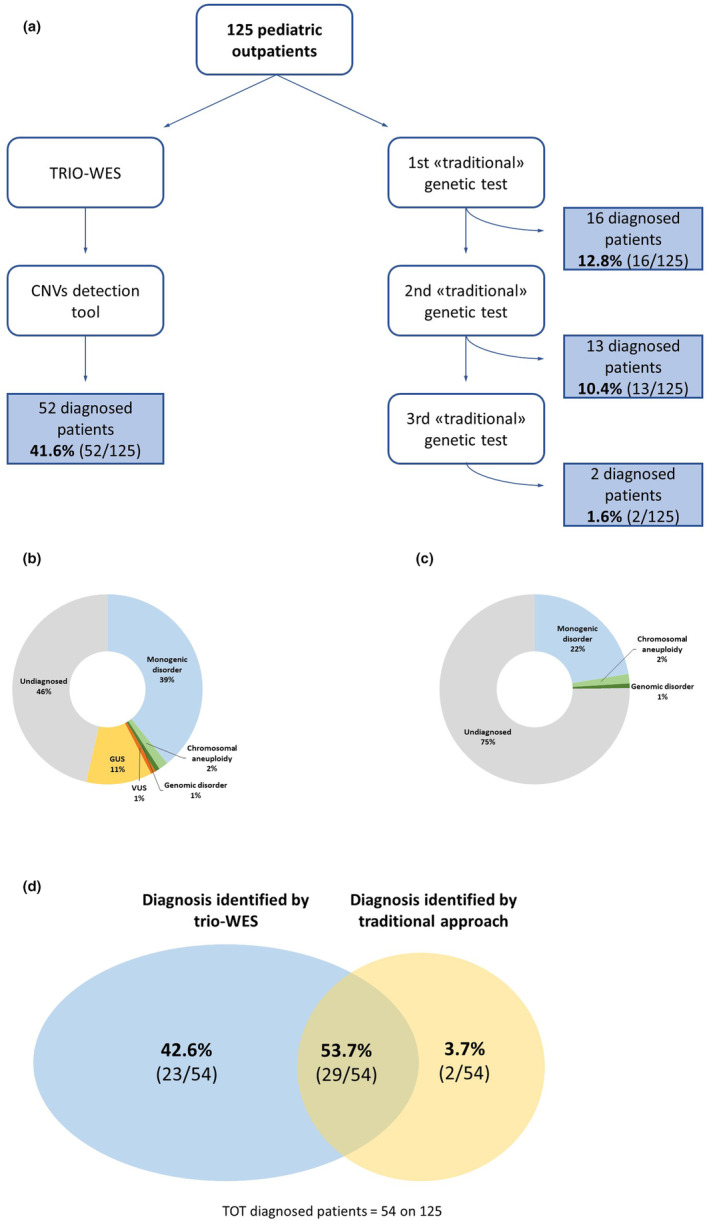
Project GENE method and results. A flowchart of the project is shown (a), with the percentage and type of diagnoses and uncertain outcomes identified with first‐tier trio‐WES (b) and traditional approach (c). A comparison in terms of diagnostic rates between the two approaches is represented in (d). CMA, chromosomal microarray; GUS, genes of unknown significance; VUS, variants of unknown significance.

Based on the latest ACMG recommendations (Manickam et al., 2021), genome‐wide sequencing tests, such as whole‐exome or whole‐genome sequencing (i.e. WES/WGS), must be performed as first‐ or at least second‐tier test in patients with a suspected genetic condition. Although the efficacy of this approach is widely demonstrated in the literature, these guidelines are far from being applied to non‐urgent patients in real‐world settings. Consequently, many outpatients often face a long and costly ‘odyssey’ to reach the correct diagnosis. Our study compared a first‐tier trio‐WES approach with a multistep ‘conventional’ one on 125 subsequent outpatients with a suspected genetic condition in terms of diagnostic yield and effectiveness (Figure 1). As a direct consequence of the inclusion criteria, our study showed an underrepresentation of critically ill patients or patients with complex malformation syndromes who usually undergo genetic testing prenatally or immediately after birth, as well as patients with metabolic diseases detected by Extended Newborn Screening (Ruoppolo et al., 2022); in contrast, cases with neurodevelopmental disorders were more prevalent as they received genetic evaluation later and in a non‐urgent setting. Consequently, a limitation of our study is that it does not comprehensively represent the paediatric genetic population.

The diagnostic rate of trio‐WES in association with a CNV‐calling tool as first‐tier test in our cohort was 41.6%, perfectly in line with the average of 30%–46% reported by recent reviews (Manickam et al., 2021; Srivastava et al., 2019). Such trio‐WES DR turned out to be considerably higher than those observed with the traditional approach. Through the second one, in fact, only about 25% of the probands reached a diagnosis, particularly half of them with the first proposed test and the others by a second or third one. These data are also in accordance with meta‐analyses that estimate a DR of a classic diagnostic pathway ranging from 7% (for metabolic testing, single gene testing) to 25% (with CMA and gene panels) (Manickam et al., 2021; Shickh et al., 2021).

Notably, the study resulted in 44% of WES‐reached diagnoses that would be missed with the traditional approach. This means that first‐tier trio‐WES has enabled the avoidance of a lengthy diagnostic odyssey in about half of the diagnosed patients, being a major aid for tailoring the correct management and follow‐up of these patients and saving time and resources. The molecular characterization has also strong implications for the patient's family, especially for the right assessment of parents' reproductive risk and for providing correct prenatal options.

On the contrary, three patients (thus, 2.4% of the cohort) were diagnosed out of the trio‐WES approach: two of them showed, respectively, a pathogenic nucleotide repeat expansion of *FMR1* and a low mosaicism (9%) for trisomy 8 on fibroblasts. The third patient had a MidXq28‐duplication syndrome (OMIM #300815) inherited from the mother: such microduplication was not detected by the exome‐based CNV‐tool because of the missing information of a mild disability in the patient's mother. The conditions unseen by trio‐WES point out the well‐known intrinsic limitations of NGS techniques (in these cases, for the detection of nucleotide expansions and low levels of mosaicism) (Marwaha et al., 2022), along with the paramount importance of a proper and deep parental and family history investigation in order to maximize the diagnostic yield of wide genome analysis.

The DRs of the two diagnostic approaches varied in relation to the phenotypic class of referral and, consequently, to the proportion of cases with a defined diagnostic suspect: in our cohort about 31% of patients were enrolled with a diagnostic hypothesis and only 33% of these suspects were confirmed. For the most represented classes in our cohort, malformative pictures and skeletal dysplasias were those with a higher percentage of suspected patients; however, even among these classes, trio‐WES outperformed the traditional pathway in terms of DR (about 55% and 46.7% for first‐tier WES vs. 44.8% and 26.7% for classical pathway respectively). The clinical heterogeneity and ultra‐rarity of many genetic conditions together with atypical, nonspecific and composite phenotypes make it difficult to establish an accurate diagnostic suspicion most of the times (Rosina et al., 2022) and particularly for neurodevelopmental disorders, whose differential diagnosis is complicated by a high extent of phenotypic overlap and a constantly increasing number of known causative genes (Sánchez‐Luquez et al., 2022). In our cohort, the positive rate of trio‐WES in NDDs was considerably higher compared to the traditional approach (36.5% vs. 13%). Therefore, our results are consistent with those of previous studies that evidenced WES as a highly effective first‐tier diagnostic tool in NDDs patients, with a yield of 30%–43% (Smith et al., 2019; Srivastava et al., 2019).

Interestingly, trio‐WES analysis detected variants in candidate GUS genes in 19.4% of cases; in particular, two‐thirds of these were identified in patients with NDDs phenotype. One of the identified GUS, *PPP1R12A*, successively has been confirmed as disease‐gene of Genitourinary and/orbrain malformation syndrome (OMIM #618820) (Hughes et al., 2020), while a recent publication correlates *SYNCRIP* mutations in a new neurodevelopmental disorder (Gillentine et al., 2021). These data once again highlight the power of WES/WGS analyses in identifying novel and ‘potential’ diagnosis that would be loss with a traditional approach. This is especially true in NDDs patients, for which targeted panels are based only on known disease genes and cannot be updated on a daily basis (Srivastava et al., 2019).

The improving DR due to the incorporation of exome‐based CNV calling in our study was only of 0.8% (1 on 125), largely under‐represented in comparison to the 6%–18% reported across literature (Posey et al., 2017; Royer‐Bertrand et al., 2021; Zhai et al., 2021). Nevertheless, these estimates are affected by the size and typology of the analysed cohort: our study excluded a priori patients who carried out CMA in prenatal or neonatal age and so a large proportion of patients for whom this examination is more frequently diagnostic, such as cases with multiple congenital anomalies and/or with a clinical picture strongly suggestive of a distinct microdeletion/microduplication syndrome. This is supported by the fact that, although CMA was performed as first/second ‘traditional’ test in 61% of patients, pathogenic CNVs were found in only 2.5% of them, against the 10%–15% DR estimated for CMA in children population (Clark et al., 2018; Miller et al., 2010). Thus, a unifying test (that could identify SNVs and CNVs) might indeed avoid misattribution of the clinical picture to CNVs that are frequent in the population and characterized by incomplete penetrance, whose findings often lead to premature termination of the diagnostic process (Maya et al., 2020; Royer‐Bertrand et al., 2021).

Ascertained the DR advantage, the aspects that might lean towards the choice of a traditional approach over first‐tier WES could be the referral time and costs. A precise comparison concerning these issues is beyond the scope of this paper. However, our study points out that, in laboratories with a dedicated team and workflow, trio‐WES analysis can be performed with a time‐to‐result of about 50 days even in outpatients setting, comparable to that of most genetic tests and certainly inferior to a pathway of iterative genetic testing (Klau et al., 2022). In addition, the per‐patient costs of WES have been greatly decreasing over time: several international studies have performed cost–benefit analyses, demonstrating that early implementation of ‘wide’ analysis is convenient also at the economic level (Ontario Health (Quality), 2020; Stark et al., 2017). In fact, a rapid diagnosis, but also the concurrent exclusion of several genetic conditions, greatly impact on patient's care and spare unnecessary medical procedures (Manickam et al., 2021). Finally, if we look at the Italian context, based on a loco‐regional healthcare system, the uneven resources cause different access to WES for non‐urgent patients, and consequently an inequality in diagnosis and treatment (Landi et al., 2021).

Our study is not without limitations: first, the small number of enrolled patients did not allow statistical data about DR of the two approaches according to the phenotypic classes and age groups. Moreover, our cohort does not fully represent the genetic paediatric population because of the aforementioned exclusion criteria, as well as the recruiting process (e.g. a high proportion of skeletal dysplasia cases is explained by the presence of two reference centres for these conditions as sending hospitals).

In summary, our results provide further evidence of how first‐tier genome‐wide sequencing tests, such as WES, significantly improve the diagnostic yield for paediatric outpatients with a suspected underlying genetic aetiology, thereby changing the current diagnostic algorithm. The ‘wide’ and agnostic approach is particularly suitable for those conditions with high aspecificity and genetic heterogeneity, like neurodevelopmental disorders, which affect most of the paediatric outpatients accessing WES. It is therefore possible that, with the increasing availability of scientific/bioinformatic resources, WGS could replace WES as first‐line diagnostic test, overcoming its technical pitfalls and still increasing the detection yield.

## AUTHOR CONTRIBUTIONS

Maria Iascone conceived the research, made substantial scientific contributions, carried out WES analyses and revised the paper. Lidia Pezzani contributed to the study design, data collection and major revision of the manuscript. Erica Rosina drafted and revised the manuscript. Erika Apuril was involved in data collection and in writing the original manuscript. Laura Pezzoli, Daniela Marchetti and Camilla Lucca performed trio‐WES analyses, data curation and validation of WES results. Matteo Bellini performed the bioinformatic analysis of the WES data. Angelo Selicorni, Maria Francesca Bedeschi, Luigina Spaccini, Donatella Milani, Anna Cereda, Lidia Pezzani, Lorenzo Colombo, Silvia Maitz, Elisa Cattaneo, Agnese Scatigno, Milena Mariani, Marta Massimello and Camilla Meossi were involved in the recruitment of the patients, the data collection, the discussion about genetic tests' results and the genetic counselling to the families. All authors have read and agreed to the published version of the manuscript.

## CONFLICT OF INTEREST STATEMENT

The authors declare no conflict/competing interests.

## ETHICAL APPROVAL

The study was complied with the Declaration of Helsinki and was approved by the Ethics Committee of ASST Papa Giovanni XXIII of Bergamo (Code: 30/2019; date: 14 February 2019) as part of the GENE Project.

## Supporting information


Supplementary Table S1.

## Data Availability

The data that support the findings of this study are available from the corresponding author upon reasonable request.
