# Supplementary material for: Comparison of first‐tier whole‐exome sequencing with a multi‐step traditional approach for diagnosing paediatric outpatients: An Italian prospective study
Source: Mol Genet Genomic Med. 2023 Dec 2;12(1):e2316. doi: 10.1002/mgg3.2316 (PMC10767581; doi:10.1002/mgg3.2316)
Supplement: Supplementary file 1 — Supplementary Table S1. [file MGG3-12-e2316-s001.docx]

| Supplementary Table 1: Clinical data and molecular results of trio-WES diagnosed patients. | | | | | | |
| --- | --- | --- | --- | --- | --- | --- |
| **N° CASE** | **SEX** | **AGE** | **PARENTAL CONSANGUINITY** | **CLINICAL PICTURE** | **DIAGNOSIS** | **MOLECULAR MECHANISM** |
| 3 | M | 10 years 2 months | No | Macrocephaly, Chiari I malformation, axillary lipoma, intestinal polyposis, pigmented macules of the glans penis | PTEN hamartoma tumor syndrome OMIM #158350 **AD** | *PTEN* NM_000314.6:c.361_362dup p.Ile122fs, htz  class 4 ***de novo*** |
| 5 | F | 1 year | No | Developmental delay, hypotonia, macrocephaly, dysmorphisms | Intellectual developmental disorder OMIM #616355 **AD** | *PPP2R5D* NM_006245.3:c.1258G>A p.Glu420Lys, htz class 5 ***de novo*** |
| 7 | F | 2 years 8 months | No | Macrocephaly, disproportionate short stature, limb shortness, metaphyseal and vertebral anomalies, bilateral Madelung's anomaly | Pseudoachondroplasia OMIM #177170 **AD** | *COMP* NM_000095.3:c.1045G>T p.Asp349Tyr, htz class 5 ***de novo*** |
| 8 | M | 10 years | No | Intellectual disability, epilepsy, microcephaly, dysmorphisms, overweight | Microcephaly, short stature, and impaired glucose metabolism OMIM #616033 **AR** | *TRMT10A* NM_152292.5:c.379C>T p.Arg127ter, hmz class 5 **mat/pat** |
| 10 | M | 2 years 4 months | No | Wide anterior fontanelle, myoclonic seizures, dysmorphisms, aortic coarctation, VSD | KBG syndrome OMIM #148050 **AD** | *ANKRD11* NM_013275.6:c.1903_1907del p.Lys635fs, htz class 5 ***de novo*** |
| 11 | M | 5 years 7 months | No | Congenital heart defects, mesocardia, recurrent cough | Ciliary dyskinesia, primary, with or without situs inversus OMIM #608644 **AR** | *DNAH5* NM_001369.3:c.5563dup p.Ile1855fs, htz class 5 **mat** NM_001369.3:c.10615C>T p.Arg3539Cys, htz class 5 **pat** |
| 13 | M | 3 months | No | Dysmorphisms, preauricular tags, left hand hexadactyly, bifid thumb, imperforate anus | Townes-Brocks syndrome OMIM #107480 **AD** | *SALL1* NM_002968.3:c.824del p.Leu275fs, htz class 4 ***de novo*** |
| 15 | F | 1 year | No | Dysmorphisms, nonobstructive ventricular hypertrophy, sucking difficulties, poor growth | Noonan syndrome OMIM #163950 **AD** | *PTPN11* NM_002834.5:c.328G>A p.Glu110Lys, htz class 5 ***de novo*** |
| 16 | F | 1 month | No | Dysmorphisms, growth retardation, hypothyroidism | Turner syndrome **Chromosomal disorder** | 45,X |
| 19 | M | 3 months | No | Pre- and postnatal growth retardation, developmental delay, dysmorphisms, hoarse cry, bilateral sensorineural hearing loss, brachydactyly, single transverse palmar creases, proximally placed thumbs, cutis marmorata | Cornelia de Lange syndrome OMIM #122470 **AD** | *NIPBL* NM_133433.4:c.7790T>C p.Leu2597Pro, hzt class 4 ***de novo*** |
| 20 | M | 6 months | No | Bone expansive lesions at the distal end of the radius and tibia, bilaterally | Caffey disease OMIM #114000 **AD** | *COL1A1* NM_000088.4:c.3040C>T p.Arg1014Cys, htz class 5 **pat** |
| 21 | F | 3 years 2 months | No | Macrocephaly with scaphocephaly, motor impairment, hypotonia, ventriculomegaly, dysmorphisms, V finger clinodactyly | Intellectual developmental disorder with autism and macrocephaly OMIM #615032 **AD** | *CHD8* NM_001170629.2:c.5422C>T p.Arg1808ter, htz class 4 ***de novo*** |
| 23 | M | 5 years 2 months | No | Short stature, skeletal dysplasia with positive maternal history | Geleophysic dysplasia OMIM #614185 **AD** | *FBN1* NM_000138.5:c.5183C>T p.Ala1728Val, htz class 5 **mat** |
| 24 | F | 3 years 5 months | No | IUGR, developmental delay, microcephaly, dysmorphisms | Intellectual developmental disorder OMIM #614104 **AD** | *DYRK1A* NM_001396.5:c.349C>T p.Arg117ter, htz class 5 ***de novo*** |
| 27 | F | 1 year 5 months | No | Short limb dwarfism | Diastrophic dysplasia OMIM #222600 **AR** | *SLC26A2* NM_000112.4:c.835C>T p.Arg279Trp, hmz class 5 ***mat/pat*** |
| 28 | M | 1 months | No | Dysmorphisms, ventricular septal hypertrophy, VSD, bilateral pyelectasis, cryptorchidism | Noonan syndrome OMIM #163950 **AD** | *PTPN11* NM_002834.5:c.1391G>C p.Gly464Ala, htz class 5 ***de novo*** |
|  |  |  |  |  | Klinefelter syndrome **Chromosomal disorder** | 47,XXY |
| 33 | M | 2 years 10 months | Yes, not specified | Skeletal dysplasia | Spondyloepimetaphyseal dysplasia, Shohat type OMIM #602557 **AR** | *DDRGK1* NM_023935.3:c.406G>A p.Glu136Lys, hmz class 4 **mat/pat** |
| 34 | F | 4 years 2 months | No | Developmental delay, dysmorphisms, ASD, VSD, pectus excavatum | KBG syndrome OMIM #148050 **AD** | *ANKRD11* NM_013275.6:c.2366_2369del p.Lys789fs, htz class 4 ***de novo*** |
| 35 | F | 7 months | No | Growth retardation, hypertonia, microcephaly, dysmorphisms, gastroesophageal reflux | Cornelia de Lange syndrome OMIM #300882 **XLD** | *HDAC8* NM_018486.3:c.697G>T p.Asp233Tyr, htz class 4 ***de novo*** |
| 37 | M | 4 years 8 months | No | Moderate developmental delay, generalized epilepsy | Developmental and epileptic encephalopathy OMIM #617711 **AD** | *PPP3CA* NM_000944.5:c.1340-1G>C, htz class 4 ***de novo*** |
| 38 | M | 16 years 9 months | No | Limited range of elbow motion, hypoevoked patellar reflexes, nail dystrophy, flatfeet | Nail-patella syndrome OMIM #161200 **AD** | *LMX1B* NM_002316.4:c.745C>T p.Arg249ter, htz class 5 ***de novo*** |
| 43 | F | 7 years 3 months | No | Intellectual disability, epilepsy, dysmorphisms, strabismus, hypermetropia, DM type I, overweight | Congenital disorder of glycosylation, type IIn OMIM #616721 **AR** | *SLC39A8* NM_022154.5:c.610G>T p.Gly204Cys, htz class 5 **mat** NM_022154.5:c.1166A>G p.Asn389Ser, htz class 4 **pat** |
| 46 | F | 1 years 7 months | No | Microcephaly, axial hypotonia, developmental delay, dysmorphisms, bicuspid aortic valve, congenital knee dislocation, camptodactyly | Trisomy 9p **Genomic disorder** | chr9:(32384599-38622233)x3 [GRCh37/hg19] |
| 47 | M | 16 years | No | Borderline intellectual disability, dysmorphisms, severe micrognathia, conductive hearing loss, scoliosis | Cerebrocostomandibular syndrome OMIM #117650 **AD** | *SNRPB* NM_198216.1:c.308dup p.Gly105fs, htz  class 4 ***de novo*** |
| 49 | M | 7 months | No | Developmental delay, hypotonia, dysmorphisms, POF | Mitochondrial complex IV deficiency, nuclear type 2 OMIM #604377 **AR** | *SCO2* NM_005138.3:c.418G>A p.Glu140Lys, htz class 5 **pat**  NM_005138.3:c.512G>A p.Arg171Gln, htz class 5 **mat** |
| 51 | M | 4 months | Yes, first cousins | Plagiocephaly, arthrogryposis, multiple fractures, clubfeet | Bruck syndrome OMIM #259450 **AR** | *FKBP10* NM_021939.4:c.975del p.Met326fs, hmz class 4 **mat/pat** |
| 53 | F | 3 years 1 months | No | Severe developmental delay, absent speech, hypotonia | Developmental and epileptic encephalopathy OMIM #613721 **AD** | *SCN2A* NM_021007.3:c.60_61delinsC p.Glu30fs, htz class 4 ***de novo*** |
| 57 | M | 5 years 5 months | No | Developmental delay, absent speech, hypotonia, macrocephaly, EEG anomalies, nystagmus, retinopathy, dysmorphisms | Intellectual developmental disorder OMIM #614104 **AD** | *DYRK1A* NM_001396.5:c.1764del p.His590ter, htz class 4 ***de novo*** |
| 60 | F | 3 years 3 months | No | Ectodermal dysplasia, 2nd-3th/4th-5th toes syndactyly, supernumerary nipple | Ectodermal dysplasia-syndactyly syndrome OMIM #613573 **AR** | *NECTIN4* NM_030916.3:c.1117C>T p.Arg373ter, hmz class 4  **mat/pat** |
| 63 | F | 9 years 5 months | No | Typical dysmorphisms, short stature, microcephaly | Myhre syndrome OMIM #139210 **AD** | *SMAD4* NM_005359.6:c.1499T>C p.Ile500Thr, htz class 5 ***de novo*** |
| 64 | F | 1 months | No | Developmental delay, hypotonia, growth retardation, dysmorphisms, micrognathia, cleft palate, arthrogryposis | Trisomy 13 **Chromosomal disorder** | 47,XX +13 |
| 67 | F | 6 months | No | Cerebellar dysplasia, oculomotor apraxia, mild hypotonia | Poretti-Boltshauser syndrome OMIM #615960 **AR** | *LAMA1* NM_005559.4:c.1034del p.Gln345fs, htz class 4 **mat** NM_005559.4:c.3038_3039del  p.Glu1013fs, htz class 4 **pat** |
| 70 | M | 6 months | No | Microcephaly, bilateral choanal atresia, mandibular hypoplasia, dysmorphisms | Mandibulofacial dysostosis, Guion-Almeida type OMIM #610536 **AD** | *EFTUD2* NM_004247.4:c.1297_1298del p.Met433fs, htz class 5 ***de novo*** |
| 72 | M | 4 years 3 months | No | Severe language delay, microcephaly, dysmorphisms | Developmental and epileptic encephalopathy OMIM #617665 **AD** | *YWHAG* NM_012479.4:c.619G>A p.Glu207Lys, htz class 4 ***de novo*** |
| 73 | M | 3 years 1 month | No | Developmental delay, epilepsy, hypotonia | KBG syndrome OMIM #148050 **AD** | *ANKRD11* NM_013275.6:c.2408_2412del p.Lys803fs, htz  class 5 ***de novo*** |
| 74 | M | 3 years 10 months | No | Mild developmental delay, dysmorphisms, right hydroureteronephrosis, hypoplastic fifth fingernail, 4th-5th toe syndactyly | Cerebellar dysfunction with variable cognitive and behavioral abnormalities OMIM #614756 **AD** | *CAMTA1* NM_015215.4:c.2863C>T p.Arg955Trp, htz class 4 ***de novo*** |
| 78 | M | 7 months | No | Aspecific brain anomalies, preauricular tags, anal atresia | Vissers-Bodmer syndrome OMIM #619033 **AD** | *CNOT1* NM_016284.5:c.7011C>G p.Phe2337Leu, htz class 4 ***de novo*** |
| 85 | M | 1 year 10 months | No | Developmental delay, hypotonia, ataxic gait, dysmorphisms, VSD, pectus carinatum, pyloric stenosis | Developmental and epileptic encephalopathy OMIM #300607 **XL** | *ARHGEF9* NM_015185.3:c.347G>A p.Arg116His, hemizygous class 4 **mat** |
| 86 | M | 2 years 7 months | No | Cystic hygroma, developmental delay, dysmorphisms, VSD, hands and feet polydactyly | Intellectual developmental disorder OMIM #615761 **AD** | *SETD5* NM_001080517.3:c.922C>T p.Arg308ter, htz class 4 ***de novo*** |
| 89 | F | 3 years 7 months | No | Developmental delay, epilepsy, ataxia, tremors, strabismus, dysmorphisms | GLUT1 deficiency syndrome 2, childhood onset OMIM #612126 **AD** | *SLC2A1* NM_006516.4:c.1129G>C p.Ala377Pro, htz class 4 ***de novo*** |
| 90 | F | 5 months | No | Pre- and postnatal growth retardation, developmental delay, dysmorphisms, VSD | KBG syndrome OMIM #148050 **AD** | *ANKRD11*  NM_013275.6:c.6938_6941dup  p.Ala2315fs, htz class 4 ***de novo*** |
| 91 | M | 7 months | No | Hypotonia, growth retardation, dysmorphisms, sparse and steely hair, hypopigmented forelock, horseshoe kidney, reduced levels of serum copper and ceruloplasmin | Menkes disease OMIM #309400 **XLR** | *ATP7A* NM_000052.7:c.1870-515_2626+1976dup 8-12 exons duplication, hemizygous class 5  **mat** |
| 98 | M | 11 months | No | Developmental delay, brain anomalies, dysmorphisms, hypospadia, talipes calcaneovalgus | Sifrim-Hitz-Weiss syndrome OMIM #617159 **AD** | *CHD4*  NM_001273.5:c.3433G>A p.Asp1145Asn, htz  class 4 ***de novo*** |
| 101 | M | 2 years | Yes, distant | Developmental delay, behavioral disorders, hypotonia | Developmental and epileptic encephalopathy OMIM #613721 **AD** | *SCN2A* NM_021007.3:c.2614_2615dup p.Ile873fs, htz class 4 ***de novo*** |
| 102 | F | 4 months | No | Hyperlactacidemia, axial hypotonia and peripheral hypertonia, severe developmental delay | Spinocerebellar ataxia 42, early-onset, severe, with neurodevelopmental deficits OMIM #618087 **AD** | *CACNA1G* NM_018896.5:c.623T>C p.Leu208Pro, htz class 5 ***de novo*** |
| 112 | F | 2 years 11 months | No | Motor developmental delay, dysmorphisms, severe joint hyperlaxity, soft and velvety skin | Loeys-Dietz syndrome OMIM #615582 **AD** | *TGFB3* NM:003239.5:c.926G>A p.Arg309His, htz class 4 ***de novo*** |
| 113 | F | 1 year 3 months | No | Epilepsy, accessual episodes of psychomotor slowdown, thinning of the corpus callosum, umbilical hernia | PCWH syndrome OMIM #609136 **AD** | *SOX10*  NM_006941.4:c.422T>G P.Leu141Arg, htz class 4 ***de novo*** |
| 114 | M | 6 years | No | Developmental delay, microcephaly, dysmorphisms | Neurodevelopmental disorder with dysmorphic facies and distal limb anomalies OMIM #617755 **AD** | *BPTF* NM_182641.4:c.1365_1370delinsCATTCC p.Arg457Pro, htz class 4 ***de novo*** |
| 118 | M | 2 years | No | Prenatal growth retardation, severe harmonic short stature, delayed tooth eruption | Rothmund-Thomson syndrome, type 2 OMIM #268400 **AR** | *RECQL4* NM_004260.4:c.213G>T p.Glu71Asp, htz class 4 **mat** NM_004260.4:c.901G>T p.Glu301ter, htz class 4 **pat** |
| 121 | F | 8 years 9 months | Yes, first cousins | Borderline intellectual disability, bilateral congenital cataract, syndactyly, diabetes mellitus type 1, benign chronic leuko-neutropenia | Wolfram syndrome OMIM #222300 **AR** | *WFS1* NM006005.3:c.2140A>G p.Asn714Asp, hmz  class 4 **mat/pat** |
| 122 | F | 4 months | Yes, first cousins | Arthrogryposis, delayed femoral head ossification, hypo- and hyperpigmented spots | Hyaline fibromatosis syndrome OMIM #228600 **AR** | *ANTXR2* NM_058172.6:c.994del p.Leu332fs, hmz class 5 **mat/pat** |
| 125 | F | 14 years 2 months | No | Mild intellectual disability, dysmorphisms, ptosis and strabismus, brachydactyly, hypertrichosis, trichotillomania and emotional disorder | Weiss-Kruszka syndrome OMIM #618619 **AD** | *ZNF462* NM_021224.6:c.2253_2254dup p.Pro752fs, htz  class 5 ***de novo*** |
| 108 | M | 5 years | No | Developmental delay, unilateral VII cranial nerve palsy, VSD, severe overweight and overgrowth, positive family history for epilepsy in mother and brother | Skraban-Deardorff syndrome OMIM #617616 **AD** | *WDR26* NM_025160.7:c.1217A>G p.Tyr406Cys, htz class 3 **mat** |
|  |  |  |  |  |  |  |
| AD: autosomal dominant; AR: autosomal recessive; ASD: atrial septal defect; EEG: electroencephalogram; hmz: homozygous; htz: heterozygous; IUGR: intra uterine growth restriction; mat: maternal; pat:paternal; POF: patent foramen ovale; VSD: ventricular septal defect; XL: X-linked; XLR: X-linked recessive | | | | | | |
